# Failure of an Innovative Low-Cost, Noninvasive Thermotherapy Device for Treating Cutaneous Leishmaniasis Caused by *Leishmania tropica* in Pakistan

**DOI:** 10.4269/ajtmh.19-0430

**Published:** 2019-10-07

**Authors:** Suzette Kämink, Ahmed Abdi, Charity Kamau, Shakil Ashraf, Muhammad Asim Ansari, Naveeda Akhtar Qureshi, Henk Schallig, Martin P. Grobusch, Jena Fernhout, Koert Ritmeijer

**Affiliations:** 1 Médecins Sans Frontières, Quetta, Pakistan;; 2 Department of Infectious Diseases, Amsterdam University Medical Centers, Center of Tropical Medicine and Travel Medicine, University of Amsterdam, Amsterdam, The Netherlands;; 3 Médecins Sans Frontières, Amsterdam, The Netherlands;; 4 Mohtama Shaheed Benazir Bhutto Hospital, Quetta, Pakistan;; 5 Department of Animal Sciences, Parasitology Laboratory, Quaid-i-Azam University, Islamabad, Pakistan;; 6 Department of Medical Microbiology, Parasitology Unit, Amsterdam University Medical Centers, Amsterdam, The Netherlands

## Abstract

Cutaneous leishmaniasis (CL), a neglected parasitic skin disease, is endemic in Pakistan, where *Leishmania tropica* and *Leishmania major* are the causative protozoan species. Standard treatment with antimonial injections is long, painful, and costly; has toxic side effects; and is not always available in public hospitals. Small pilot studies have previously evaluated a low-cost and noninvasive hand-held exothermic crystallization thermotherapy for cutaneous leishmaniasis (HECT-CL) device. We aimed to further establish the effectiveness, safety, and feasibility of HECT-CL in *L. tropica*. In a prospective observational study, patients with parasitological confirmation of CL were treated using the HECT-CL heat pack for 3 minutes with an initial temperature of 52–53°C for 7 consecutive days. Dried blood spot samples were taken for species identification by polymerase chain reaction (PCR). Effectiveness was assessed by using medical photographs and measurements of the lesion size at baseline and subsequent follow-up visits, for up to 180 days. We intended to enroll 317 patients. The HECT-CL treatment was easy to apply and well tolerated. Species identification demonstrated the presence of *L. tropica*. Interim analysis of 56 patients showed a failure rate of 91% at follow-up (median 45 days after treatment, interquartile range 30–60 days). Enrollment of patients was prematurely suspended because of futility. This study showed a high failure rate for HECT-CL thermotherapy in this setting. *Leishmania tropica* is known to be less sensitive to antileishmanial drugs, more temperature-resistant, and spontaneous healing is slower than that in *L. major*. More research is needed to identify low-cost, effective, and more patient-friendly treatment for *L. tropica.*

## INTRODUCTION

Cutaneous leishmaniasis (CL) is a neglected tropical disease, which is caused by different species of the protozoan genus *Leishmania.* Globally, CL affects 1 to 1.5 million people, mainly in the Middle East, Central Asia, North Africa, and Latin America.^1–5^ Cutaneous leishmaniasis is endemic in Pakistan, and the incidence is rising, especially in Balochistan, Punjab, and northeastern regions.^3,6–8^ As CL is not a notifiable disease in most provinces of Pakistan, cases are likely to be underreported, and the estimated annual incidence is 22,000–36,000 cases.^1,3,6,7^ Untreated CL lesions are a reservoir for further transmission of CL in the community, often causing complications with secondary infections and ulcerations, which leave disfiguring scars, and psychosocial suffering as a consequence, due to the stigma and social isolation.^9–11^

In Pakistan, CL is transmitted by phlebotomine sand flies, and the causative parasitic *Leishmania* species are *Leishmania tropica* and *Leishmania major*. *Leishmania tropica* is spread anthroponotically in the highlands and urban areas. *Leishmania major* is transmitted zoonotically by mammals such as gerbils and jirds as the main reservoir in lowland, rural, and (sub)urban settings.^2,5,8,12–16^

The incubation time of CL is between 2 and 8 months.^2^ Cutaneous leishmaniasis often starts with a papule that develops into a nodule or ulcerating lesion, depending on several factors, such as *Leishmania* species, virulence, and immune response of the host.^2,5,17,18^ The *L. major* lesions are usually self-healing within 3–6 months, yet leaving behind a depressed scar. In *L. tropica*, it takes typically 1 year or more to heal, and infected lesions often develop into an unremitting, chronic form, if left untreated, which can take up to several years to heal without treatment.^2,16^ In Pakistan, meglumine antimoniate (Glucantime^™^, Sanofi-Aventis, France) is the mainstay of treatment for CL. Meglumine antimoniate is registered in the country only since April 2018 and until date not available in government hospitals. The WHO provides Glucantime to the Ministry of Health in Pakistan on an irregular basis. Consequently, antimonial products of unknown quality and even counterfeits are available on the local market at high prices and often administered by unqualified staff who do not know the correct treatment course. Since 2008 until date, Médecins Sans Frontières (MSF) is the only health-care provider who effectively treats patients with CL, free of charge.

Antimonial treatment is administered by injections in 70% of the cases by biweekly painful intralesional injections, for 4 to 6 weeks. In the other 30%, systemic treatment is required, consisting of daily intramuscular injections (20 mg antimony/kg/day) for 20 days, which is costly and leads to general adverse effects such as muscle pain, fever, nausea, and abdominal pain, and it has the potential to elicit organotoxic adverse effects, such as cardiomyopathy, nephropathy, pancreatitis, and hepatic complications.^2,16^ Furthermore, the high financial burden of the lengthy treatment for patients and their families (transportation costs and loss of income due the frequent visits) is another reason for high treatment attrition rates.

Over the past decades, several alternative treatment options have been studied. Thermotherapy (TT) is a potential option, as *Leishmania* amastigotes appeared thermosensitive.^19–23^ Thermotherapy is often applied by the ThermoMed^®^ instrument, manufactured by Thermosurgery Technologies Inc., Phoenix, AZ It is a portable, battery-operated, radio frequency generator. Thermotherapy modalities have shown good efficacy with short healing time; however, the effectiveness depends on the *Leishmania* species, population, and country.^20–22,24–26^ In Old World CL (OWCL), the reported cure rate of *L. tropica* with radio frequency TT varied between 54% and 94%.^20,22,27^ The disadvantage is that the application with the ThermoMed is very painful. For this reason, lidocaine 2% solution has to be injected around the lesions, before the TT session; otherwise the pain becomes unbearable for the required duration of 30 seconds. Also, secondary burns usually occur. Furthermore, the initial investment is between 5,000 and 10,000 USD, and the ThermoMed has high maintenance costs, which are possibly prohibitive in contexts where CL occurs.

Based on the principle of heat sensitivity of the *Leishmania* parasite, in recent years, a simpler mode to deliver external heat to the skin has been evaluated for the treatment of CL, which is easier in use, cheaper, and less painful: the hand-held exothermic crystallization thermotherapy for cutaneous leishmaniasis (HECT-CL) (Pristech Products Inc., San Antonio, TX).^28,29^ The HECT-CL is a heat pad, a polyvinyl chloride (PVC) bag of 10 × 7 cm^2^ that contains a solution of sodium acetate (3H2O CH3COONa) in water with a ferrous flexible disk of 2 cm ø inside; bending of this disk initiates crystallization, leading to the release of heat. The saturation of the solution is calibrated to produce 52°C (±2°C) for about 3 minutes.^28,29^ The cost of one HECT-CL pack is less than two USD, and it is reusable by heating up in boiled water. The HECT-CL is easy to carry, to use, and to store.

The HECT-CL has been evaluated in a few small pilot studies; one conducted in Peru, and one in Pakistan (1, 2). Both studies concluded that TT with HECT-CL has good efficacy, has definite cure with complete re-epithelialization of the lesions, and is safe, and the side effects in the two studies were generally mild. The heat pad, applied on the lesion, heats up the skin which is assumed to kill the *Leishmania* parasites, which are harbored at the surface of the skin. In the 29 patients studied in the Peruvian study, the efficacy of the HECT-CL showed 63% per protocol (PP) (52% intension-to-treat [ITT]). In the study in Pakistan, with 27 participants, the efficacy was 83% PP (70.4% ITT). In both studies, the cohort size was small, and the proportion of patients lost to follow-up was relatively high. The Peruvian study concerned CL caused by New World cutaneous *Leishmania* species (*L. braziliences*, *L. guyanensis*, and *L. peruviana*). In the study in Pakistan (Sindh), the causative OWCL species were not determined, but the predominant *Leishmania* species in Sindh is *L. major.*^6,7,13,14,30,31^

In this prospective observational study, we aimed to establish evidence of effectiveness, safety, and feasibility of the HECT-CL heat pack. The low-cost HECT-CL device, provided it has an acceptable effectiveness and good tolerability, can potentially be rolled out to peripheral health facilities as an alternative to treatment with antimonial injections, which would have a major impact on access to CL treatment.

## METHODS

### Study design and patients.

The study is a prospective observational cohort study in which a single cohort of CL patients is treated with HECT-CL and followed up for 6 months. The study population is the CL patients who present at the MSF-supported Mohtarma Shaheed Benazir Bhutto Hospital in Quetta, Pakistan.

Inclusion criteria were CL skin lesions of less than 6 months duration, with diagnostic confirmation of CL by microscopic visualization of *Leishmania* amastigotes in a Giemsa-stained fine needle aspirate (FNA), and written informed consent from patients aged ≥ 18 years or from a legal guardian if a patient was < 18 years old.

Exclusion criteria were patients younger than 10 years, lesions located on or within 2 cm between the eyes and lips, more than four lesions, lesions with a diameter (ø) of more than 6 cm, pregnant and lactating women < 6 months after delivery, patients with uncontrolled medical illnesses, immune disorders such as HIV/AIDS, or receiving steroid medication.

### Diagnosis.

The diagnosis of CL was established by a dermatologist’s physical examination and laboratory confirmation by identification of *Leishmania* amastigotes in an FNA from the raised edge of the ulcer. Dried blood spot (DBS) samples were taken for species identification using PCR at the Quaid-i-Azam University (QAU), Islamabad. Dried blood spot were also sent for quality control to the Academic Medical Centre, Amsterdam, Netherlands.

### Treatment.

The treatment was provided by a trained health worker and consisted of pressing the HECT-CL heat pack firmly on the lesion, covering the entire lesion surface, including the induration area, for 3 minutes for 7 consecutive days. The initial surface temperature of the HECT-CL device was 52–53°C. The temperatures of the HECT-CL device and of the skin were measured with an infrared thermometer (General Tools IRT207 Laser Temperature Gun, Thermal Detector, 8:1 Mid-Range Infrared Thermometer, Secaucus, NJ) before and after treatment.

### Sample size.

To estimate the effectiveness at an expected level of 75% and with a precision of 5% at a 95% probability (α = 0.05) and a power of 80% (β = 0.2), a sample size of 288 with known outcome at 180 days was required. Because that in the regular program, the defaulter rates are low (0.5%) and adherence to follow-up visits is high, the expected lost to follow-up was estimated at a maximum of 10% of patients; therefore, the final sample size was calculated for 317 CL patients.

### Outcome parameters.

#### Effectiveness.

Photographs and measurements of the lesions taken at baseline and on the five follow-up visits (day 15, 30, 60, 90, and 180) were used to compare lesion evolution with baseline data.

Clinical characteristics were used to evaluate the effectiveness of the HECT-CL.^2,16,32,33^ Lesions were monitored by repeated photographs and measurements.

The “initial treatment response” was defined as complete re-epithelialization of all ulcers and complete disappearance of induration < 45 days after the treatment start.^16,33^ “Definite cure” was defined as appearance of the characteristic scarring tissue (hyper- or hypo-pigmented) and complete re-epithelialization of the treated lesion, flattening, and absence of super infections 180 days after the HECT-CL treatment start. “Treatment failure” was defined as 1) nodule, plaque, or ulceration, which has increased on day 15 and/or on day 30 compared with day 0 (at baseline); 2) no improvement observed on day 60 compared with day 0; 3) less than 50% of the lesion re-epithelialized or flattened, and persistent signs of inflammation, or increase on day 60; and 4) less than 100% re-epithelialized or flattened ≥ day 90 and ≤ 180 days with parasitological confirmation.^16,28,32,33^

#### Safety.

Safety was evaluated by occurrence of any adverse event as mentioned by the patient and/or observed by the examiner during or up to 30 days after treatment (e.g., edema, pain, and burns were graded for severity) and by the proportion of occasions in which the HECT-CL application was too painful and the procedure had to be interrupted or topical lidocaine 5% was required.

#### Feasibility.

Feasibility was evaluated by the ease of use by the health worker, the acceptability by patients, and the (in) appropriateness of the treatment, that is, the percentage of initially screened patients with locations or lesion characteristics where HECT-CL could be used, but IL treatment could not (e.g., joints, too large), or HECT-CL could not be used (e.g., near the eyes and on the lips).

### Follow-up procedure.

During the follow-up visits, the lesions were measured and photographed for monitoring the healing progress.

### Other definitions.

A “defaulter” was defined as a patient who did not finish the seven treatment sessions. A “no show” was defined as a patient who did come to one of the scheduled follow-up visits on days 15, 30, 60, or 90 after active trace finding. Patients were considered “lost to follow-up” when they did not come to the final follow-up visit.

### Rescue treatment.

Rescue treatment with the routine therapy of intralesional or intramuscular meglumine antimoniate injections was offered in case of treatment failure.

### Statistical analysis.

#### Data and statistical analysis.

The data were analyzed by descriptive statistics. The composition and proportions of the different variables, gender, age, town, province, type of lesion, location of lesion, size (ø), and previous CL treatment, were described. The different categorical (dichotomous and nominal) and continuous variables of the anthropometrical measurements and demographic characteristics were summarized using the number of patients (*n*) and percentage (%) and/or median and interquartile range (IQR). To analyze safety, the initial temperature of the heat pack, the grade of severity, and the therapy session day (day 0–6) at which the adverse event or serious adverse event had occurred were recorded.

### Ethics.

This study was approved by the MSF Ethics Review Board and the Pakistan Health Research Council; ClinicalTrials.gov number: NCT03208543.

## RESULTS

**Figure 1.**
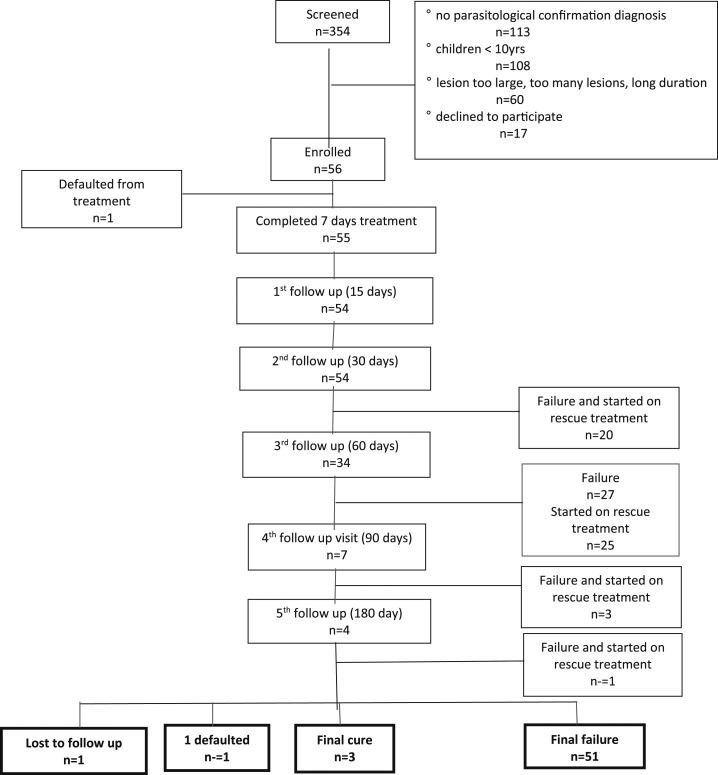
Study flow chart.

In the first 2 months of the study, 354 CL patients presented at the hospital, of whom 56 patients (15.8%) were recruited for the study. The other 298 CL patients (84.2%) were not eligible as they did not meet the inclusion criteria (Figure 1).

### Diagnosis.

In all 56 enrolled patients, the diagnosis of CL was established by physical examination by a dermatologist and parasitological confirmation in an FNA smear. Thirty DBS samples (DBS) were randomly taken for species identification using PCR to the QAU laboratory in Islamabad, of which 22 samples were identified as *L. tropica*; eight samples contained DNA material insufficient to perform *Leishmania* species identification. Of these 30 samples, seven were sent to the Amsterdam University Medical Centers (AUMC) parasitology laboratory in Amsterdam for quality control, which confirmed the presence of *L. tropica* in all seven samples.

### Patient characteristics.

Among the 56 patients, 23 were male (41.7%) and 33 were female (58.9%). The median age of the patients was 20 years (IQR 12.5–35) for both males and females.

On presentation at the hospital, the median duration of the lesions was 3 months (IQR 2–3.5). All 56 patients together had 88 lesions: 35 patients (62.5%) presented with one lesion, 12 patients (21.4%) with two lesions, seven patients (12.5%) with three lesions, and two patients (3.6%) with four lesions. Of the 88 lesions, 16 (18.1%) were on the face, 12 (13.6%) were located on the arms, nine (10.2%) were on the wrist, 10 (11.4%) were on the hands, 13 (14.8%) were located on the fingers, eight (9.1%) lesions were located on the legs, 18 (20.5%) were on the feet, and two (2.3%) were on the toes. Of the 88 lesions, the erythematous nodule was most common with 49 (55.7%) lesions, 30 (34.1%) were noduloulcerative lesions, seven (8.0%) were ulcers, and two lesions (2.3%) were plaques.

Of the 56 patients, one patient (1.8%) defaulted after five treatment sessions, and another patient (1.8%) was lost to follow-up (Figure 1, Table 1).

**Table 1:** Characteristics of hand-held exothermic crystallization thermotherapy for cutaneous leishmaniasis patients

Variable	*n* (%)
Gender	
Male	23 (41.1)
Female	33 (58.9)
Age (years)	
≥ 10 and < 16	20 (35.7)
≥ 16 and < 21	12 (21.4)
≥ 21 and < 40	12 (21.4)
≥ 40	12 (21.4)
Duration of lesion (months)	
1	10 (17.9)
2	11 (19.6)
3	21 (37.5)
4	6 (10.7)
5	8 (14.3)
No. of lesions per patient	
1	35 (62.5)
2	12 (21.4)
3	7 (12.5)
4	2 (3.6)
Location of the lesions	
Face	16 (18.1)
Arm	12 (13.6)
Wrist	9 (10.2)
Hand	10 (11.4)
Finger	13 (14.8)
Leg	8 (9.1)
Foot	18 (20.5)
Toe	2 (2.3)
Type of lesion	
Nodule	49 (55.7)
Plaque	2 (2.3)
Ulcer	7 (8.0)
Noduloulcerative	30 (34.1)

### Treatment.

Fifty-five patients completed the treatment with the HECT-CL for 3 minutes on 7 subsequent days. The median initial surface temperature of the HECT-CL was 52.6°C (IQR 52.5–52.8). The median temperature of HECT-CL after the 3 minutes was 48.6°C (IQR 48.4–49.0). The median skin temperature after the 3 minutes was 39.6°C (IQR 39.0–41.0).

### Outcome.

#### Effectiveness.

Two months after its start, the study was suspended because of futility. Interim analysis of the first 56 patients showed that 80.6% of the lesions increased in size (ulcer, edges, and induration) and/or deteriorated in signs of inflammation and ulceration, or no improvement was observed on day 60, according to the standard definitions for treatment failure (Table 2). Further investigations showed that there had not been a breach of standard operational procedures and no procedural errors were made. Investigators decided to stop further recruitment of participants on ethical grounds. The final cure rate was 5.4% (3/56 patients) in an intention to treat analysis. A per-protocol analysis showed a final cure rate of 5.6% (3/54) and a cumulative final failure rate of 94.4% (51/54) (Table 2). All failures were parasitologically confirmed by the presence of *Leishmania* amastigotes in the lesions.

**Table 2:** Response to hand-held exothermic crystallization thermotherapy for cutaneous leishmaniasis

Follow-up day	*n* (%)
On day 15	
Improvement	2
No improvement	28
Increased	23
No show	2
On day 30	
Improved	3
No improvement	10
Increased	19
Failure	20
No show	3
Cumulative failure	20 (35.7)
On day 60	
Improvement	2
No improvement	2
Increased	3
Failure	27
No show	1
Cumulative failure	47 (80.4)
On day 90	
Improvement	3
Failure	3
No show	2
Cumulative failure	50 (89.3)
On day 180	
Final cure	3
Failure	1
Lost to follow-up	2
Cumulative final failure	51 (91.1)

ITT analysis; *n* = 56.

**Figure 2.**
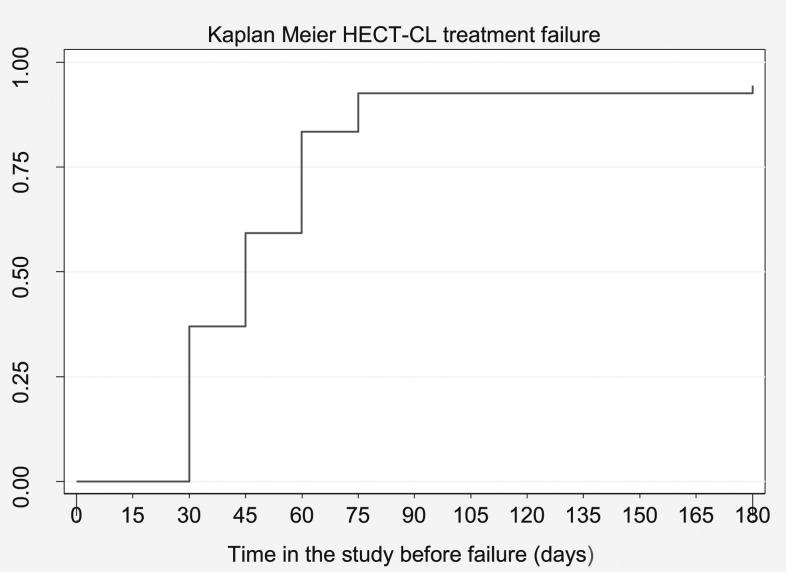
Kaplan–Meier hand-held exothermic crystallization thermotherapy for cutaneous leishmaniasis treatment failure.

Of the 56 patients, 20 patients (35.7%) had clinical failure at 30 days after treatment because the nodule, plaque, or ulceration had increased compared with photographs and measurements taken on day 0 (baseline), day 6, and day 15. At day 45, another 12 patients visited the clinic because the lesion was increasing and/or deteriorating and considered as failures (57.1%). Sixty days after the treatment start, the lesions of additional 15 patients (80.4%) had failed to heal because less than 50% of the lesion was re-epithelialized or flattened and signs of inflammation did not reduce or even increased. At day 75, three patients (89.3%) failed, and at the last follow-up day (180), another patient (91.1%) failed. Treatment failure was established at median 45 days after treatment (IQR 30–60 day) (Figures 1 and 2, Tables 1 and 2).

Of those who failed the TT, rescue treatment with meglumine antimoniate injections was offered as per protocol. Two of the 51 patients who failed treatment declined this rescue treatment; they were too scared for the injectable drugs. The other 49 patients were followed up during and after rescue treatment, and 48 were cured without further complications within 2 months after starting the meglumine antimoniate treatment. One patient’s lesion did not heal by the meglumine antimoniate. This patient had already relapsed from a previous antimonial treatment.

#### Safety.

The treatment with HECT-CL was well tolerated with a minimum of adverse events. After 3 minutes, the HECT-CL skin was slightly red, but no blistering occurred. None of the patients required local anesthesia because of intolerable pain; the patients scored an average of 2.5 on the Wong–Baker FACES Pain Rating Scale. Twelve patients (23.1%) developed secondary infections during the follow-up period.

#### Feasibility.

Health workers reported that the HECT-CL is easy to use, but time-consuming. Regarding acceptability, none of the enrolled patient discontinued the treatment because of dissatisfaction with the treatment. Four lesions treated with the HECT-CL were not appropriate for administration of the intralesional injection because of the location. Sixty of 116 (51.7%) patients who were 10 years or older with confirmed CL were not eligible for treatment with HECT-CL because of the size, number, or site of the lesions.

## DISCUSSION

The rational for this study was to establish evidence of effectiveness of the HECT-CL device in the treatment of CL in Pakistan, which could have resulted in a recommendation to roll out the HECT-CL as an effective, safe, and affordable treatment option for CL. However, the outcome of this study showed a failure rate of 94.4%. The high failure rate is in sharp contrast with the other study performed with the HECT-CL in Pakistan. The plausible reason for the failure of the HECT-CL could be the difference in *Leishmania* species, with differences in heat sensitivity and differences in spontaneous healing between *L. major* and *L. tropica*. Previous epidemiological studies have shown that western and northern provinces of Pakistan are predominately affected by *L. tropica*, whereas in the southern and eastern provinces, Sindh and Punjab, and in the semiurban and rural areas of Balochistan, *L. major* is found to be the main causative species.^6,8,13,31,34–36^ Thus, in the earlier successful pilot HECT-CL study in Sindh, the predominant species was most likely *L. major*, whereas in our study, *L. tropica* was the species identified as causative species. *Leishmania tropica* is known to be less sensitive to antileishmanial drugs and more temperature resistant.^18–21,24,37^ Second, *L. major* lesions usually heal spontaneously within 2–8 months.^2^ In the *L. major* study, the medium lesion duration of patients was 4 months (IQR 30–720 days), and the healing response occurred between 3 and 6 months after the HECT-CL treatment.^29^ Therefore, study outcomes in *L. major* may have been biased by this spontaneous healing during the follow-up period after HECT-CL therapy. In *L. tropica*, spontaneous healing can take a year or longer.^2^ In our study, the medium duration of lesions was 3 months on admission. This means that 6 months after HECT-CL treatment in *L. tropica* lesions, self-healing could have occurred in only a very small proportion of patients. This is what we have observed in our study as well.

The TT with the ThermoMed device has been found effective in *L. tropica* and is one of the therapeutic interventions recommended by the WHO to treat CL.^2,16,19,26^ This ThermoMed device delivers precisely controlled localized current field heat (50°C for 30 seconds) generated by radio frequency energy that produces 6.78 MHz frequency and penetrates deep into the skin.^20,22,23,38,39^ The temperature of the HECT-CL device in this study was 52−53°C (initial temperature) and was applied on the surface of the skin. Although tissue temperature during the treatment session could not be measured in this study, the skin temperature measured immediately after 3 minutes of HECT-CL application was < 41°C (median 39.6°C; IQR 39.0−41°C), compared with the HECT-CL device temperature after 3 minutes of 48.6°C (IQR 48.4–49.0). Therefore, skin tissue temperature during the three-minute treatment session has most likely been insufficient to achieve the isoeffective thermal dose, that is, the relationship between temperature and time of exposure has to be effective to kill the parasites or trigger an effective immune response.^40^ The fact that the same method of application was used as in the Sindh study and a similar skin temperature was measured after the application of the HECT-CL (mean skin temperature 38.7–41.8°C) further supports the argument of bias by spontaneous healing in studies with *L. major*.

Furthermore, lesions were not able to heal and re-epithelialize in this study, which resulted in a high incidence of secondary infections during follow-up 12/54 (23.1%).

The outcome of the three patients with definite cured lesions on day 180 is possibly due to spontaneous healing during the 3-month follow-up period.

The decision to treat a patient with CL is often justified to accelerate the healing process, prevent complications (secondary infections and disfiguring scars), or cure relapsing patients, and reduce disease transmission in the community.^25,41^ The current mainstay treatment is still with pentavalent antimony injections, and other evidence-based treatment modalities are needed. However, studies on other treatment options for *L. tropica* are scarce, inconsistent, or inconclusive.^42,43^ This refers to topical treatment options, such as paromomycin ointment, TT, CO_2_ laser, or cryotherapy, and other systemic treatment options, such as miltefosine, amphotericin B, or itraconazole. Some options seem promising in *L. tropica* but require more study in larger cohorts. In general, combination therapy seems to be more effective.^42,43^

## CONCLUSION

The curative effect of the HECT-CL heat pack device could not be established in CL caused by *L. tropica* in Pakistan. More research is needed to identify low-cost, effective, and more patient-friendly treatment for *L. tropica,* and more evidence needs to be established on the efficacy of HECT-CL in treatment for *L. major*.
